# Silicon-mediated Improvement in Plant Salinity Tolerance: The Role of Aquaporins

**DOI:** 10.3389/fpls.2017.00948

**Published:** 2017-06-08

**Authors:** Juan J. Rios, Maria C. Martínez-Ballesta, Juan M. Ruiz, Begoña Blasco, Micaela Carvajal

**Affiliations:** ^1^Department of Plant Nutrition, Centro de Edafología y Biología Aplicada del Segura – Consejo Superior de Investigaciones CientíficasMurcia, Spain; ^2^Department of Plant Physiology, Faculty of Sciences, University of GranadaGranada, Spain

**Keywords:** silicon, aquaporins, nutrient uptake, abiotic stress, salinity stress, water relations, water use efficiency

## Abstract

Silicon (Si) is an abundant and differentially distributed element in soils that is believed to have important biological functions. However, the benefits of Si and its essentiality in plants are controversial due to differences among species in their ability to take up this element. Despite this, there is a consensus that the application of Si improves the water status of plants under abiotic stress conditions. Hence, plants treated with Si are able to maintain a high stomatal conductance and transpiration rate under salt stress, suggesting that a reduction in Na^+^ uptake occurs due to deposition of Si in the root. In addition, root hydraulic conductivity increases when Si is applied. As a result, a Si-mediated upregulation of aquaporin (PIP) gene expression is observed in relation to increased root hydraulic conductivity and water uptake. Aquaporins of the subclass nodulin 26-like intrinsic proteins are further involved in allowing Si entry into the cell. Therefore, on the basis of available published results and recent developments, we propose a model to explain how Si absorption alleviates stress in plants grown under saline conditions through the conjugated action of different aquaporins.

## Introduction

The uptake of mineral nutrients is regulated by transporters in the root plasma membranes. In general, there is a strong interaction between the uptake of ions and water uptake, since both are dependent on each other. Therefore, the interactions between water transporters (aquaporins) and nutrients transporters need to be determined in root cells. Nutrient deprivation or excess due to changing environmental conditions usually involves fundamental parameters, including the water relations in plants, in which aquaporins play an important role. One of the first pieces of evidence regarding water-nutrient connections was found in plants deprived of nitrogen and phosphorus, in which there was a reversible reduction of cell and root hydraulic conductivity involving aquaporins (Carvajal et al., 1996). It was also reported that, when a plant is subjected to nutrient deficiency, alterations in aquaporins slow the movement of water through the plant (Clarkson et al., 2000; Shaw et al., 2002). The balance of nutrient supply received by roots can be regulated by aquaporins and ATPase and Ca-ATPase activities (Martínez-Ballesta et al., 2003; Cabañero et al., 2006). Therefore, it was suggested that aquaporins can play a central role in nutrient homeostasis, which is likely to comprise (i) support for ion fluxes through provision of an accompanying water flow and (ii) active re-direction of apoplastic/symplastic water flow within tissues and the whole plant (Maathuis et al., 2003).

Plant aquaporins belonging to the MIP (membrane intrinsic proteins) family are mainly homotetrameric transmembrane proteins that facilitate water transport through membranes, but they can also form heterotetramers (for review see Martínez-Ballesta and Carvajal, 2016). The classification of aquaporins into seven subfamilies is mostly based on phylogenetic distribution, while their localization in different membranes will be used for the nomenclature (e.g., PIP, TIP, and NIP). In addition to facilitating water diffusion, a number of aquaporins have also been shown to transport other molecules (Gerbeau et al., 1999; Bienert et al., 2011). During the last decade it was reported that aquaporins transport specific solutes, like urea (Liu et al., 2003), ammonia (Bertl and Kaldenhoff, 2007), carbon dioxide (Uehlein et al., 2003), hydrogen peroxide (Bienert et al., 2007), lactic acid (Choi and Roberts, 2007), boric acid (Takano et al., 2006), and silicic acid (Ma et al., 2006). The trafficking and subcellular relocalization of aquaporins could be the critical point in the regulation of the transport of mineral nutrients to the cytoplasm, since aquaporins are translocated from the endoplasmic reticulum (ER) to the plasma membrane via the Golgi apparatus (Maurel et al., 2009). However, the molecular and cellular mechanisms underlying the interactions of aquaporins and mineral nutrients still need to be investigated.

Silicon (Si), the second most abundant element in the earth’s crust but its essentiality in plant growth and development remains debated since plants differ widely in their ability to take up Si (Sommer et al., 2006). Silicic acid, Si(OH)_4_, is the only form known to be absorbed by plants (Ma and Yamaji, 2006). It will enter plant roots mainly by diffusion via the apoplastic pathway but requires the presence of specific aquaporins, NIP2s, to enter the symplastic pathways and be eventually translocated to aerial organs via the xylem (Guerriero et al., 2016).

Although Si is generally considered non-essential for plants, some species will accumulate between 1 and 5% on a dry weight basis. Families such as the Poaceae (grasses), most species of monocotyledons, aquatic macrophytes, and some dicotyledons, including the Cucurbitaceae (Rogalla and Römheld, 2002; Piperno, 2006; Schoelynck et al., 2012) have all been shown to accumulate high concentrations and benefit from Si presence. However, even in non-accumulating plants, the presence of Si in nutrient solutions or soils has been reported to be beneficial against abiotic stress (e.g., NaCl; for a review see Zhu and Gong, 2014), but the mechanisms of Si action in relation to water uptake and aquaporins are poorly understood. In this review, the improvement of plant salinity tolerance by Si through enhancement of root water uptake, including the regulation of aquaporin activity and gene expression, is discussed.

## Si Nutrition and Salinity Stress

Si is generally considered non-essential for plant development, but many authors consider Si a ‘quasi-essential’ element for higher plants, since plant growth may be stimulated by the supply of Si and Si-starved plants may display physical abnormalities (Rafi and Epstein, 1997; Epstein and Bloom, 2005; Ma and Yamaji, 2008). It can enhance growth, yield, and crop quality, particularly under biotic and abiotic stresses, such as herbivory, leaf microbial pathogens, UV radiation, gravity, extreme temperatures, lodging, metal toxicity, nutrient deficiency and toxicity, drought, and salinity (Epstein, 1994, 1999, 2009; Ma and Takahashi, 2002; Richmond and Sussman, 2003; Cooke and Leishman, 2011; Van Bockhaven et al., 2013; Liang et al., 2015).

Salinity stress is an important factor that limits crop yields and productivity worldwide, affecting approximately 800 million hectares (ha) of arable land (FAO, 2008). Although our understanding of the role of Si in abiotic stress resistance is still limited, important advances with regard to salinity stress have been made (Rengasamy, 2010). In fact, it has been widely reported that the provision of Si increases salt tolerance and hence biomass in many important crops grown under different conditions, such as barley (Liang et al., 2005a), wheat (Tuna et al., 2008; Ali et al., 2012; Bibordy, 2014), rice (Gong et al., 2006; Mahdieh et al., 2015), soybean (Lee et al., 2010) sugarcane (Ashraf et al., 2010), tomato (Romero-Aranda et al., 2006; Muneer et al., 2014; Li et al., 2015), and cucumber (Khoshgoftarmanesh et al., 2014), among others.

### Na^+^ and K^+^ Homeostasis

At high salt concentrations, one of the main salt-tolerance mechanisms is the maintenance of low intracellular Na^+^ concentration by the reduction of Na^+^ influx and/or the increase of Na^+^ efflux. Na^+^ enters roots passively, via non-selective cation channels and trough other Na^+^ transporters such as HKT (high-affinity K^+^ transporter) family; consequently, Na^+^ is critical to maintain intracellular K^+^ concentration (Blumwald, 2000; Munns and Tester, 2008; Kronzucker et al., 2013). It has been shown that Si may alleviate salinity stress by affecting Na^+^ and K^+^ concentrations (Ashraf et al., 2010). They found interactive effects of NaCl, Si, and genotype, on Na^+^, K^+^, and the K^+^/Na^+^ ratio (a salt-stress indicator) in sugarcane. In this study, the addition of Si reduced Na^+^ uptake and transport to the shoots and increased the shoot K^+^ concentration, with a resultant increase in the K^+^/Na^+^ ratio. Similarly, Xu et al. (2015) and Garg and Bhandari (2016) reported that Si application to salt-stressed aloe plants, and sensitive and tolerant genotypes of *Cicer arietinum* L., significantly decreased the Na^+^ content in roots and its translocation to leaves, while improving K^+^ uptake, consequently raising the K^+^/Na^+^ ratio.

### Effects on Nutritional Balance

It is important to point out that one of the main deleterious effects of salinity is an imbalance in essential nutrients. Recent studies on the plant ionome have shown that salinity causes modifications of the tissue levels of macronutrients like N, Ca, P, S, and Mg, and micronutrients such as Zn, Mn, Fe, and B. Hellal et al. (2012) reported increased N, P, and Ca concentrations in the shoots and seeds of fava bean grown under salt stress when Si was supplied. Similarly, Si enhanced the P, Ca, and Mg contents in leaves and roots of aloe and tomato plants (Li et al., 2015; Xu et al., 2015), and maintained higher P and Fe contents in salt-stressed canola plants (Farshidi et al., 2012). Application of Si significantly increased the Ca concentration in shoots of cucumber plants exposed to salinity, while it had no effect on the shoot Ca concentration of plants grown under non-saline conditions (Khoshgoftarmanesh et al., 2014). By contrast, the supply of Si decreased the S content in *Zinnia elegans* exposed to salinity stress. However, the salinity-induced reduction of micronutrients such as Zn, Mn, Fe, and B was alleviated by Si addition (Manivannan et al., 2015). In previous reports, NaCl stress was found to increase Cu levels in several plant species (Wang and Han, 2007), but in the study by Manivannan et al. (2015), the level of Cu was not affected. These studies provided evidence that Si might induce salt tolerance in many crops, not only via inhibition of Na^+^ uptake and translocation, and improvement of the plant K^+^ content, but also by affecting the plant status of some other essential nutrients in order to maintain normal physiological conditions. A summary of the relationships between Si and different inorganic ions in plants grown under salinity stress is shown in **Table 1**.

**Table 1:** Summary of the relationship between Si and different inorganic ions in plants subjected to salt stress.

Inorganic ions	Relation	Plant species	Reference
Na	Antagonism	*Saccharum officinarum* *Aloe vera* *Cicer arietinum*	Ashraf et al., 2010 Xu et al., 2015 Garg and Bhandari, 2016
K	Synergism	*Saccharum officinarum* *Aloe vera* *Cicer arietinum*	Ashraf et al., 2010 Xu et al., 2015 Garg and Bhandari, 2016
N	Synergism	*Vicia faba*	Hellal et al., 2012
P	Synergism	*Vicia faba* *Aloe vera* *Solanum lycopersicum*	Hellal et al., 2012 Xu et al., 2015 Li et al., 2015
Ca	Synergism	*Vicia faba* *Aloe vera* *Solanum lycopersicum Cucumis sativus*	Hellal et al., 2012 Xu et al., 2015 Li et al., 2015 Khoshgoftarmanesh et al., 2014
Mg	Synergism	*Vicia faba* *Ale vera*	Hellal et al., 2012 Xu et al., 2015
S	Antagonism	*Zinnia elegans*	Manivannan et al., 2015
Zn	Synergism	*Zinnia elegans*	Manivannan et al., 2015
Mn	Synergism	*Zinnia elegans*	Manivannan et al., 2015
Fe	Synergism	*Zinnia elegans* *Brassica napus*	Manivannan et al., 2015 Farshidi et al., 2012
B	Synergism	*Zinnia elegans*	Manivannan et al., 2015
Cu	No relation	*Zinnia elegans*	Manivannan et al., 2015

### Protection from Oxidative Damage

Plants produce low levels of reactive oxygen species (ROS), which form part of the chemical communication in cells. However, salinity also inhibits plant growth via an overproduction of ROS that can damage macromolecules essential for plant growth and development, such as DNA or lipid membranes. It has been demonstrated recently that Si mitigates oxidative stress by stimulation of antioxidants, both enzymatic and non-enzymatic (Savvas and Ntatsi, 2015), such as superoxide dismutase (SOD), catalase (CAT), ascorbate peroxidase (APx), peroxidases (POD), glutathione (GSH), and ascorbate (AA). Many authors have reported the beneficial effects of Si with regard to amelioration of salt-induced oxidative stress. Li et al. (2016) showed that the provision of Si in Hoagland’s solution at 1, 2, 4, or 6 mM increased the POD activity of *Glycyrrhiza uralensis* seedlings grown under salt stress, after 20 days of treatment. In this study, SOD activity was intensified only at 4 mM Si and the malondialdehyde (MDA) concentration was significantly decreased at all Si levels, compared with the saline control (50 mM NaCl). Garg and Bhandari (2016) showed that the oxidative markers O_2_^-^, H_2_O_2_, and MDA were more abundant in *Cicer arietinum* genotypes subjected to long-term salinity, but their levels declined when 4 mM Si was supplied. Additionally, SOD, CAT, guaiacol peroxidase (GPOX), APx, monodehydroascorbate reductase (MDHAR), dehydroascorbate reductase (DHAR), and AA were increased in salt-stressed plants of both genotypes by Si supplementation. Likewise, Li et al. (2015) reported increased MDA and H_2_O_2_ concentrations and decreases in SOD and CAT activities in salt-stressed tomato seedlings grown under sand culture; however, Si application reversed all these stress-induced changes. In contrast, Bibordy (2016) found that SOD and CAT activities were suppressed by the supply of Si (2 or 4 g L^-1^) to canola plants grown under saline conditions. Although differing plant responses to salt stress have been demonstrated, Si supplementation, generally, seems to lead to a decline in ROS production and an increase in ROS scavenging enzymes and antioxidant compounds. Hence, at the cellular level, Si might ameliorate salinity-induced oxidative stress due to more efficient use of ROS-scavenging metabolic pathways, which may increase membrane integrity. This also might be related with a better Na^+^-K^+^ cellular status and an improvement of the plant ionome.

### Photosynthesis and Osmoregulation

Salt stress decreases the physiological cell activities involved in photosynthesis (Garg and Bhandari, 2016), mostly due to osmotic stress, nutritional imbalance, and/or nutritional toxicity combined with later oxidative stress. However, recent evidence indicates that Si influences photosynthesis through effects on water uptake and transport. Mateos-Naranjo et al. (2013) showed that the negative effect of high salinity on gas exchange, water-use efficiency (WUE), pigment concentrations (Chl*a* and Chl*b*), and PSII efficiency, was reversed by Si supply for the halophytic grass *Spartina densiflora*. On the other hand, Abbas et al. (2015) reported that Si application enhanced the stomatal conductance, transpiration rate, number of stomata, and stomatal size in salt-sensitive and salt-tolerant okra plants. A complementary protective mechanism of plants growing under saline conditions is the synthesis and accumulation of different osmolytes and compatible solutes. Although this is dependent on the plant species, Si has been found to enhance the contents of proline (Tuna et al., 2008; Soylemezoglu et al., 2009; Siddiqui et al., 2014), soluble protein (Li et al., 2015), polyamines (Wang S. et al., 2015; Yin et al., 2016), glycine betaine, total free amino acids, soluble sugars, and phenolic compounds (Abbas et al., 2015).

In summary, the potential correlation between the application of Si and benefits for plants under saline conditions are: (i) maintenance of the status of essential nutrients, by reduction of Na^+^ content and improvement of K^+^ content, (ii) greater efficiency of ROS-scavenging metabolic pathways and (iii) increase of gas exchange. All these mechanism are related to water relations and water-use efficiency as it will be reviewed as follows.

## Effect of Si On Water Uptake and Transport

In accumulating species, Si has been assigned an unspecific function in crop protection, since it seems to be involved in structural and dynamic aspects of plant responses that help diminish the deleterious effect (Epstein, 2001). In fact, it is generally agreed that the positive effects of Si are more manifest under conditions of stress. For example, Yeo et al. (1999) indicated that Si could decrease Na uptake by plants under salinity stress. Studies with toxic metals such as Al indicated that silicified tissues may give protection against these metals through co-deposition of Al with Si in some monocotyledons (Sangster et al., 2001).

Accumulation of Si could occur actively (Liang et al., 2005a; Rains et al., 2006) or passively, the latter depending on the transpiration rate as described formerly (Takahashi et al., 1990). However, there are some plants that are excluders (Henriet et al., 2006; Carey and Fulweiler, 2014). The Si/Ca ratio has been reported to be indicative of the Si uptake mechanism (Carey and Fulweiler, 2014): ratios exceeding 1 indicate active uptake, ratios of 0.5–1 suggest passive uptake, and ratios below 0.5 could show exclusion. Also, another indicator proposed is the relationship between the Si availability around the root and the Si concentration inside the plant (Carey and Fulweiler, 2014). However, these indicators could change under saline conditions that alter Ca uptake and transpiration.

Recent studies have clearly established that Si uptake in plants is dependent on an influx channel-type transport, the Lsi1 channel, responsible for Si movement from the external solution into the internal cells. The first Si transporter was identified in rice (Ma et al., 2006), and subsequent studies have shown that this transporter was present in all Si-accumulating species including monocots such as barley, wheat, and maize (Yamaji et al., 2008, 2012; Chiba et al., 2009; Mitani et al., 2009; Montpetit et al., 2012) – and dicots, such as cucumber, pumpkin and soybean (Mitani-Ueno et al., 2011; Deshmukh et al., 2013; Wang H.S. et al., 2015). In addition, another transporter found in rice (Ma et al., 2007) and in a few other species, termed Lsi2, acts as an active efflux transporter carrying Si to the xylem (Ma and Yamaji, 2015). It is important in the long-distance transport of Si through the plant. Much less is known about the nature and properties of Lsi2s and they have only been described so far in monocotyledons and horsetail (Vivancos et al., 2016). Although, in general, Lsi1 and Lsi2 are localized in the plasma membrane, the distribution differs among species; in fact, in rice they are found in the exodermis and endodermis in the mature regions of the main and lateral roots (Ma et al., 2006), while in other monocotyledons such as maize and barley they are localized in epidermal, hypodermal, and cortical cells (Chiba et al., 2009; Mitani et al., 2009). In dicots, the pumpkin CmLsi1 is found in all root cells (Mitani et al., 2011) and the cucumber, CsLsi1 has been recently localized in endodermal and cortical cells in root tips and in root hairs (Sun et al., 2017). Therefore, the localization of Si transporters in the roots could be an important factor that determines how Si influences water uptake and, therefore, the sensitivity of the plant to salinity.

Silicon has been described as a protective element against abiotic stress like salinity, on the basis of its induction of changes in lignin and suberin processing and deposition, which reduces the rates of water loss and evapotranspiration (Cruz et al., 1992; Sonobe et al., 2009; Amin et al., 2016). Along the same lines, Si has been reported to increase lignification in sorghum, thereby increasing xylem resistance to water loss (Hattori et al., 2005). Some studies have also suggested that Si could induce a thicker cuticle in leaves of rice and sorghum, reducing stomatal conductance and decreasing water loss through the epidermal layer and thus maintaining the water potential in leaves (Matoh et al., 1991; Hattori et al., 2005). Furthermore, Si has been reported to improve the regulation of stomatal opening, although the mechanism behind this has not been resolved (Gao et al., 2006). Also, Gao et al. (2004) found that Si increased the water use efficiency in maize due to induction of root hydraulic conductance.

Other results have shown that Si improved the response to abiotic stress when water availability was reduced. In sorghum plants for instance, when Si was applied to the nutrient solution, there was an increase of water uptake and water flow from roots to leaves, together with an increase of stomatal conductance (Sonobe et al., 2009). Hattori et al. (2008) indicated that Si application enhanced root hydraulic conductance, and Sonobe et al. (2009) suggested that the improvement of this parameter could occur in a radial direction in the root (by modification of osmotic characteristics or expression of aquaporins) rather than axially (via modification of the number or diameter of xylem vessels). Therefore, the influence of Si on transpiration and its role in the physiology of the stomata are controversial (Agarie et al., 1998; Gao et al., 2006). It is clear that, under water deficiency, if Si only reduced transpiration, an increase in water use efficiency followed by protection against wilting would occur (Gao et al., 2004). However, if transpiration is increased, accompanied by higher root hydraulic conductance (Sonobe et al., 2009), the water use efficiency will also increase. The role of Si in water relations thus seems to be associated with the maintenance of water use efficiency, but at the moment there is not enough evidence to propose a model that clarifies this response. Therefore, future studies should focus on this matter.

**FIGURE 1:**
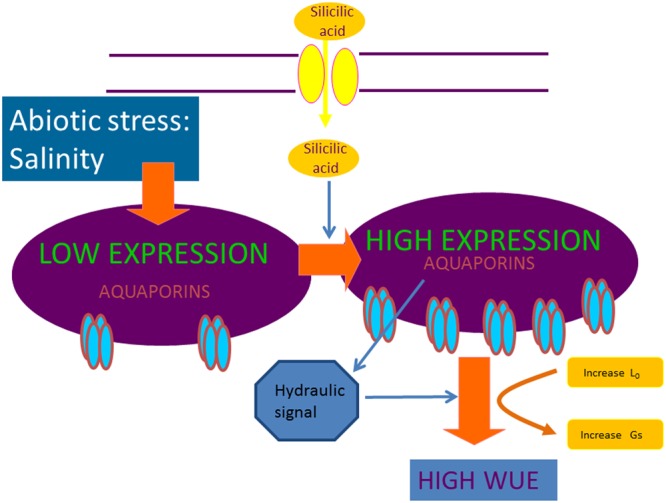
Under salinity stress, Si has been reported to improve stomatal functioning and enhance root hydraulic conductance. The physiological mechanism could involve hydraulic signaling through aquaporin expression, leading to higher water-use efficiency.

The link between water stress and Si has been well studied in tomato where Romero-Aranda et al. (2006) showed that Si enhanced drought resistance in tomato plants as a result of an increase in leaf water content. Shi et al. (2016) showed that the effects of Si not only increased root hydraulic conductance in tomato, but also maintained membrane integrity and protected it against oxidative damage by an increase of the antioxidant metabolism. Furthermore, several studies on Si and drought stress in different species, including sorghum, maize, and tomato, concluded that Si alleviated the effect of the stress (Agarie et al., 1998; Hattori et al., 2008; Sonobe et al., 2009; Shi et al., 2016). However, although insights into its mechanism of action have not been provided yet, the fact that all reported results concluded that water relations are improved, supports the notion that aquaporins and hydraulic signals are involved (**Figure 1**).

## Aquaporins and Si

Aquaporins belong to the major intrinsic protein (MIP) family and allow the transport of water and small solutes through biological membranes (Chrispeels and Maurel, 1994; Kruse et al., 2006). In plants, they are classified into different subfamilies: the plasma membrane intrinsic proteins (PIPs), the tonoplast intrinsic proteins (TIPs), the nodulin26-like intrinsic proteins (NIPs), the small basic intrinsic proteins (SIPs), the uncategorized intrinsic proteins (XIPs), the GIPs, and the hybrid intrinsic proteins (HIPs) (Danielson and Johanson, 2008), according to their subcellular localization, function, sequence length, and substrate selectivity (for review, see Maurel et al., 2015).

Aquaporins have been reported to transport distinct types of substrates, such as ammonia, antimony, arsenite, boron, carbon dioxide, formamide, glycerol, hydrogen peroxide, lactic acid, silicon, and urea (Bienert and Chaumont, 2011; Hove and Bhave, 2011). The substrate selectivity regarding the transported molecule is determined by two factors: the NPA motifs, responsible for proton exclusion, and the aromatic/arginine (ar/R) region, that functions as the main filter in the pore (Wu and Beitz, 2007). Because of this, MIPs facilitate the transport of the widest range of solutes, including several metalloids. Different isoforms belong to the NIP subfamily: the NIP1 subgroup, that is more permeable to water and glycerol, the NIP2 subgroup, that transports metalloids and is the only aquaporin subgroup able to transport Si (Mitani-Ueno et al., 2011), and the NIP3 subgroup, that is notable for its biological function in boric-acid transport. All subgroups are permeable to formamide (Dean et al., 1999; Wallace and Roberts, 2005).

It has been reported that Si plays an important role in the mechanisms that enable plants to cope with biotic and abiotic stresses (Vivancos et al., 2016). However, the capacity to transport Si depends on the plant genotype. A mechanism combining efflux (Lsi2) and influx (Lsi1) Si transporters has been reported to regulate Si accumulation in different cell compartments and plant organs and tissues (Deshmukh et al., 2015). Lsi1, a NIP2 homolog aquaporin and Si-influx transporter, was first identified in rice (Ma et al., 2006), and is conserved among different plant species. Also in rice, NIP2;2 (Lsi6) was classified as a Si transporter which enables silicic acid to pass from the xylem to leaves (Yamaji et al., 2008). Furthermore, NIP2 aquaporins have been identified only in plants where Si has a beneficial role in plant nutrition.

A peculiarity of NIPs that transport Si is their expression profile, in which they are situated on the distal side of the root endodermis plasma membrane (Ma et al., 2006; Chiba et al., 2009; Mitani et al., 2009; Yamaji and Ma, 2009). This allows cooperation with other Si transporters, such as the active Lsi2 efflux transporters, situated on the proximal side of the membrane. It has been postulated that this cell polar localization of NIPs takes place when these aquaporins have a direct role in Si uptake and translocation (Pommerrenig et al., 2015). Therefore, localization of Si transporters on different sides of the cell membrane may allow optimization of the directional Si flux. Deshmukh et al. (2015) determined that the ability to transport Si was determined by a GSGR amino-acid motif in the selectivity filter of the NIP subfamily, and when this amino-acid group was confined within a specific distance from the NPA domains. This may explain the lack of Si accumulation in some plant species such as tomato (Deshmukh et al., 2015), where an inadequate amino-acid distance between the NPA motifs is observed (Deshmukh et al., 2015). Also, a larger constriction size in the pore of NIP2 proteins, relative to other NIP subgroups, is responsible for Si transport. Furthermore, in graminaceous plants, *NIP2:1* orthologs have been described as Si transporters involved in its distribution and reallocation within these plants (Chiba et al., 2009; Mitani et al., 2009). In horsetail (*Equisetum arvense*), one of the species in the plant kingdom that accumulates high amounts of Si, Si channels of the NIP subfamily were identified. These results point out the complexity of Si uptake and distribution in the whole plant, since the ability to take up Si does not depend solely on the aquaporins, but also on the presence of active transporters (Deshmukh and Bélanger, 2016).

A dual role of aquaporins under salt stress, in the presence of Si, can be described. On the one hand, members of the PIP subfamily may act as regulators of plant water balance, and, on the other hand, the NIP subfamily can participate in Si uptake and cell levels. However, the mechanisms by which Si alleviates salinity stress via aquaporin regulation need a deeper investigation. It has been reported that Si is able to reduce Na^+^ and Cl^-^ uptake and translocation to the shoot in barley (Liang et al., 2005b), alfalfa (Wang and Han, 2007), wheat (Tuna et al., 2008), soybean (Lee et al., 2010), and rice (Gong et al., 2006; Shi et al., 2013) under salinity. In rice, a typical Si-accumulating species, inhibition of Na^+^ and Cl^-^ accumulation by Si may not involve a reduction of the transpiration stream, since Gong et al. (2006) found that stomatal conductance and transpiration were increased. Silicon formed a physical barrier in the endodermal and exodermal Casparian bands, reducing the translocation of these ions. But, whether a similar mechanism occurs in other Si accumulators must be elucidated. In tomato, a Si-excluder, the levels of Na^+^ and Cl^-^ were maintained in the plant in the presence of Si, despite the reduction of the adverse effects produced by salinity (Romero-Aranda et al., 2006). The ameliorative effect of Si on NaCl stress has been related to osmotic stress alleviation, with the involvement of aquaporins as regulators of plant water status, rather than a palliative effect on ion toxicity (Liu et al., 2015). Therefore similar beneficial effects appear to be observed in both Si-accumulating and non-accumulating plants.

In *Sorghum bicolor* L., Si regulated the expression of the PIP aquaporins, under short-term salt-stress exposure, which restored the root hydraulic conductance, L_p_, lost due to salinity. This allowed the plants to maintain their water content and rate of photosynthesis. Silicon alleviated the osmotic effect of salinity without the appearance of symptoms of Na^+^ toxicity in the plants (Liu et al., 2014; 2015). In addition, it has been observed that salt stress may reduce L_p_ through the inactivation of aquaporins by H_2_O_2_ (Boursiac et al., 2008). Silicon may enhance L_p_ by decreasing H_2_O_2_, which affects not only expression, but also PIP activity (Liu et al., 2015). Since H_2_O_2_ promotes the internalization of PIPs from the plasma membrane under salinity (Boursiac et al., 2008), an influence of Si on PIPs trafficking cannot be discounted.

It has been reported that Si may promote the development of suberized structures in the root endodermis and exodermis (Fleck et al., 2015). Apoplastic Na^+^ transport would be thus reduced, preventing the accumulation of this ion in the plant shoot (Krishnamurthy et al., 2011). This may lead to a reestablishment of the expression of PIPs, in order to maintain the water flux through the symplastic route.

Zhu et al. (2015) observed the effect of Si on two cucumber (*Cucumis sativus* L.) cultivars under salinity. In their work, Si increased the expression of the root PIP2 subfamily and decreased the osmotic potential by an increase in the root sugar content, which favored water uptake. The authors concluded that osmotic adjustment by the plants, to acquire water under salt stress, was a mechanism initiated after Si addition that developed differentially in the two cucumber genotypes.

Members of the NIP subfamily may influence plant responses to salinity through controlled Si uptake and transport. For two varieties of rice, the expression of the *OsLsi1* gene, a NIP2 homolog, increased under salt stress, but was higher in the tolerant cultivar compared to the sensitive cultivar, inducing greater Si uptake in the former (Senadheera et al., 2009). In this case, the authors described Si accumulation via the transpiration stream as a mechanism to reduce NaCl transport to the aerial parts of the plant. However, the response of *OsLsi1* expression to the addition of Si alone was the opposite of that which was observed with salinity alone, and so the study of the combination of these two factors in species with high water demand is critical (Senadheera et al., 2009).

**FIGURE 2:**
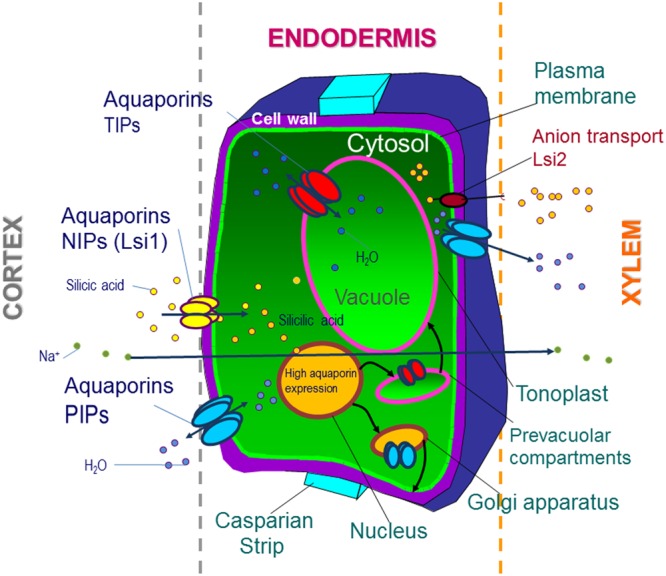
Aquaporin regulation by Si under salt stress in the root endodermis. Silicic acid enters the plant roots by water flow via the apoplastic and symplastic pathways: the symplastic pathway involves the action of aquaporins, mainly NIPs (Lsi1) localized on the distal membrane side of the efflux transporter Lsi2. NIPs allow Si accumulation in the transpiration stream, impeding Na^+^ accumulation. In addition, Si increases the expression of the root PIP aquaporin subfamily and therefore enhances root hydraulic conductance under salinity, optimizing water transport in the cell. This, together with a decreased osmotic potential of the root sap due to Si-dependent osmolyte accumulation, allows for an increase in water uptake under stress. Coordination between PIPs and TIPs is responsible for the water balance during osmotic adjustment.

Reduction of water uptake and transport induced by salinity stress appears to be alleviated by Si as a function of aquaporin activity. Indeed, NIP aquaporins promotes Si entrance into the cell, which increases the expression of root PIP aquaporin subfamily. This effect enhances root hydraulic conductance enabling an optimal water transport and reduction of Na^+^ accumulation (**Figure 2**). On the other hand, this possible mode of action is not found to be directly associated with other secondary effects such as osmotical adjustment (Pei et al., 2010; Ming et al., 2012; Liu et al., 2014) or oxidative-stress amelioration (Shi et al., 2016). Therefore, the direct role of Si on the regulation of aquaporin functionality needs further validation.

## Concluding Remarks

The evidence that Si promotes salinity tolerance via enhancement of root hydraulic conductance and water uptake, thereby contributing to increased water use efficiency, underlines the importance of studying Si uptake mechanisms and their regulation. Furthermore, if the beneficial effects of Si, in both monocotyledons and dicotyledons, are linked to the passage of water through membranes, future studies should concentrate on the influence of Si on aquaporin expression, particularly under abiotic stress conditions. Recent findings suggest that water relations involving aquaporins are the key point in the amelioration of the adverse effects of salinity stress. Considering that Si transport is also mediated by aquaporins (NIP2), this suggests that stimulation of the Si uptake system in plants could lead to a new approach to PIP aquaporin up-regulation, which in turn will reduce Na^+^ conglomeration in membranes and increase water uptake and transport. However, to further elucidate Si accumulation and understand its critical role at the whole plant level, molecular and physiological characterization of Si-transporting aquaporins in different plant species is required. The importance of the NIP2 aquaporins subgroup as Si transporters in plants highlights that aquaporins could be the subject of biotechnological intervention to produce salinity tolerant plants and cultivars biofortified with Si.

## Author Contributions

JJR, MM-B, JMR, and BB contribute to writing. MC contribute to writing, correction and production of figures.

## Funding

This work was funded by the Spanish Ministerio de Economía Industria y Competitividad (AGL2016-80247-C2-1-R). We acknowledge support of the publication fee by the CSIC Open Access Publication Support Initiative through its Unit of Information Resources for Research (URICI).

## Conflict of Interest Statement

The authors declare that the research was conducted in the absence of any commercial or financial relationships that could be construed as a potential conflict of interest.
